# Physiologically lucky: the role of medical physiology in modern medical education: An interview with Dr. John E. Hall

**DOI:** 10.1007/s40037-013-0044-5

**Published:** 2013-02-20

**Authors:** Ahmad H. Adi, Hani J. Alturkmani

**Affiliations:** Alfaisal University, College of Medicine, P.O. Box 60285, Riyadh, 11545 Saudi Arabia

**Keywords:** Physiology, Medical, Students, John E. Hall

## Introduction

As medical students, we are entrusted with the responsibility of healthcare, and thus are required to learn the fundamentals of many scientific and clinical disciplines. This includes physiology. The variety of medical subjects cannot but leave students with questions as to which is the most relevant to what they intend to pursue after their graduation; the answer to this question differs from one medical speciality to the other. We had the pleasure of interviewing Dr. John E. Hall, Chairman of the Department of Physiology at the University of Mississippi Medical Center and author of the world famous ‘Guyton and Hall Textbook of Medical Physiology’. The interview took place at the University of Mississippi Medical Center in Jackson, Mississippi, USA in August 2011. Keeping it to a medical student perspective, we discussed Physiology in the context of medical education: its current and future importance, as well as some advice from Dr. Hall to the current generation of medical students.

## Biography

Dr. John E. Hall, author of the world famous ‘Guyton and Hall Textbook of Medical Physiology’, began college with a major in Journalism and English but had a change of heart during his second year when he became more interested in science and physiology. After graduating from Kent State University in 1968 he spent two years in the army and then pursued graduate studies at Michigan State University, where he became interested in cardiovascular physiology, mathematical modelling, systems analysis and their application to physiology. He received a Ph.D. in physiology in 1974 and then worked with Dr. Arthur Guyton as a postdoctoral fellow at the University of Mississippi Medical Center, where he was promoted to faculty in about 6 months and to full professor in 6 years. In 1989 he became Chairman of the Department of Physiology when Dr. Guyton retired. He also serves as Associate Vice Chancellor of Research. Dr. Hall has published more than 500 papers in peer-reviewed journals, as well as 18 books including the ‘Textbook of Medical Physiology’. He has received many awards for his research and has served as President of the American Physiological Society, the Inter-American Society of Hypertension, and the Council for High Blood Pressure Research of the American Heart Association, as well as holding leadership positions in many other professional organizations. 

**Ahmad: You did a lot of work, is there a secret to it? Is there something that you found very helpful?**

Hall: An important aspect of success in any field is to find something that you really love. If you really love it, it’s not work, and if it’s not work, you don’t really have to be driven to do it every day; you want to get going early, and it’s difficult to go home every night, because you enjoy what you’re doing. Second, take your briefcase home every night. By this I mean that you should be reading, studying and thinking about your ‘work’ beyond the usual daily schedule. Often good ideas occur when you are not at work. The third thing is that you should feel ‘lucky’. By this I mean that you should have a very positive approach towards research and life. Sometimes you get results that are not anticipated and you may initially be disappointed. However, if you have a positive attitude, you see things from a different perspective and can sometimes find a novel and important, although unexpected, result. People who feel lucky usually are ‘lucky’.

**Ahmad: How relevant is physiology, as a basic science, to clinical practice? Do you need to be well aware of physiology to be a good doctor?**

Hall: Every year the American Association of Medical Colleges asks graduating medical students about how well the basic sciences have prepared them for clinical training, and every year physiology is at the top. Therefore, graduating medical students believe that physiology is highly relevant and important to their clinical training.

**Ahmad: the most famous thing that medical students know you about is the book, the fact that you were the co-author of the book and now you are the author of the Textbook of Medical Physiology. Do you actually know everything in your book?**

Hall: I hope so! But do I know everything about physiology? No. Physiology is a rapidly evolving science and we are always learning new things about how the body functions. I get letters almost every week from people all over the world who send me ideas about new aspects of physiology that they think I should add to the book. The Textbook of Medical Physiology, however, is not designed for research scientists; it is designed to be a teaching tool and must not exceed too much what can be taught in medical school. So whenever I add something new to the textbook, I also take something out so that the volume of material does not grow too large for the students to use effectively. Therefore, I am always faced with the challenge of determining what new material should be added and what should be removed even though the amount of knowledge about the physiology of the human body is rapidly expanding. I always appreciate feedback from students, professors and clinicians about changes in the book that they believe will make it more useful. However, I always try to keep in mind that the book is written for students, not for research scientists.

**Ahmad: How would you describe your relationship with Dr. Guyton, both professionally and non-professionally? What did he like to do outside the lab, what do you like to do outside the lab?**

Hall: Dr. Guyton was a great mentor for me. His approach was to help trainees identify important questions and whenever I proposed an experiment he would usually ask ‘why is that important?’ He was also an inspiring role model outside the lab and I was privileged to know Dr. Guyton for thirty years. He had ten children, all successful physicians. He enjoyed sailing but, except for his family, he loved physiology the most. Most of my ‘fun’ also revolves around research and physiology. I do, however, enjoy sports and I like to go boating in my spare time.

**Ahmad: how do you see physiology in the next 100** **years? What are the hot topics? How do you expect it to change?**

Hall: Physiology will continue to be an exciting field of research for a long time but I can’t predict scientific advances for the next 100 years. One example of a physiology frontier is brain function and how it integrates other systems of the body. We have a basic understanding of how various components of the nervous system work, but we don’t know nearly as much about their coordinated interactions that permit us to perform daily functions like walking, talking, and thinking in a complex manner. There are many other areas that can be considered as ‘frontiers’ for physiology research. We will have increasingly sophisticated tools that will permit advances in genetics, in vivo imaging (including molecular imaging), tissue engineering and organ regeneration, and others that will be the basis for many new advances in physiology and medicine. The great beauty of physiology is that it seeks to integrate the individual functions of the cells, tissues and organs into an understanding of the function of the entire human body, which is much more than the sum of its parts. Life relies on carefully controlled interactions of the body’s systems and understanding these complex systems is one of the great challenges for physiologists.

## Conclusion

Physiology, like many other medical fields, is a discipline that is changing as new discoveries are made. This interview only presents an example of how the changes in this field can be incorporated into modern teaching of medicine, as Dr. Hall explains about the process of editing ‘Guyton and Hall Textbook of Medical Physiology’. Whether future frontiers of physiology will be incorporated into medical texts in an easy-to-understand manner is yet to be known. But we can conclude that medical education is, like physiology, an ever-changing concept, and adaptation to the newest techniques and methods will be necessary in order to provide the best care by future physicians.

